# Demographic Inequalities in Mortality Due to Hepatocellular Cancer in Male Patients With Alcoholic Cirrhosis in the United States: A CDC WONDER Database Analysis

**DOI:** 10.7759/cureus.98057

**Published:** 2025-11-29

**Authors:** Fiqe Khan, Abdullah Ahmad, Meher Ayyazuddin, Abdul Rafeh Awan, Syma Arshad, Muhammad A Nadeem

**Affiliations:** 1 Department of Internal Medicine, The Brooklyn Hospital Center, Brooklyn, USA; 2 Department of Medicine, CMH Lahore Medical College and Institute of Dentistry, Lahore, PAK; 3 Department of Internal Medicine, Bayonne Medical Center, Bayonne, USA; 4 Department of Medicine and Surgery, Nishtar Medical University, Multan, PAK; 5 Department of Community Medicine, Rashid Latif Medical College, Lahore, PAK; 6 Department of Liver Transplant and Surgery, Digestive Disease and Surgery Institute - Cleveland Clinic, Cleveland, USA

**Keywords:** alcoholic liver cirrhosis, cdc wonder database, health care disparities, hepatocellular carcinoma (hcc), mortality rates

## Abstract

Background

This study aimed to examine alcoholic cirrhosis-associated hepatocellular carcinoma (HCC) mortality trends and disparities by sex and race in the United States between 1999 and 2020.

Methods

Using the CDC WONDER database, we analyzed HCC mortality in alcoholic cirrhosis patients, excluding females and individuals under 15, between 1999 and 2020. Mortality trends were stratified by age, race, and state. Temporal trends were assessed using the Mann-Kendall test, and continuous variables were compared using t-tests.

Results

During the study, 9,837 deaths were attributed to HCC with alcoholic cirrhosis, with an age-adjusted mortality rate (AAMR) of 0.338. Mortality increased significantly over time (p < 0.001). Young, older, and White men exhibited significant AAMR increases, with higher rates in older vs. younger men (p < 0.001). No racial differences were noted, while state-wise analysis showed the highest mortality rates in New Mexico, Vermont, and Oregon, and the lowest in Mississippi, Utah, and New Jersey.

Conclusions

The increasing mortality rate of HCC associated with alcoholic cirrhosis poses a significant challenge for healthcare providers and policymakers. Public education on the effects of alcohol consumption is essential to raise awareness, while further research is needed to develop and implement evidence-based strategies to address this growing health concern effectively.

## Introduction

Hepatocellular carcinoma (HCC) is the sixth most prevalent cancer globally and ranks as the third-leading cause of cancer-related deaths [1]. It typically develops in the setting of advanced fibrosis or cirrhosis. Alcohol-related liver disease (ALD) contributes to approximately 30% of global HCC cases and HCC-specific deaths [2].

Alcoholic cirrhosis is the advanced stage of ALD, marked by irreversible fibrosis and regenerative nodule formation following chronic excessive alcohol use. It progresses from steatosis and steatohepatitis and is defined by bridging fibrosis on histology and clinical features of hepatic decompensation, including portal hypertension and impaired synthetic function [3,4].

Globally, HCC mortality rates have been changing, with an epidemiological shift from traditional viral etiologies to a broader range of factors, including social determinants of health (SDOH) [5,6]. In the United States, the age-adjusted death rate from alcohol-related liver cirrhosis increased by 47.0% - from 4.3 deaths per 100,000 population in 2000 to 6.4 deaths per 100,000 population in 2019 [7]. Despite growing recognition of alcohol-associated liver disease as a major contributor to liver cancer mortality, large-scale national assessments specifically examining sex-, race-, and state-level disparities in HCC mortality attributable to alcoholic cirrhosis are lacking. To address this gap, we aimed to evaluate trends and demographic and geographic inequalities in HCC-related mortality due to alcoholic cirrhosis in the United States from 1999 to 2020, including age, race, geography, and urban/rural status, in order to encourage better surveillance of HCC and improve policies and public health strategies to monitor patients more effectively on a wider scale.

## Materials and methods

Study design and data sources

In accordance with STROBE (Strengthening the Reporting of Observational Studies in Epidemiology) guidelines for observational research, we conducted a retrospective, population-based analysis of mortality trends in HCC among individuals with alcoholic cirrhosis using publicly available data from the CDC WONDER Multiple Cause of Death (MCOD) database from 1999 to 2020 [8,9]. The database contains national mortality data derived from U.S. death certificates and includes information on both underlying and contributing causes of death. To improve reproducibility, deaths were classified as follows: HCC (ICD-10 C22.0) was required to be listed as the underlying cause of death, and alcoholic cirrhosis (ICD-10 K70.3) was required to appear as a contributing cause of death in the multiple-cause fields. This approach ensured that all included deaths represented individuals whose primary cause of death was HCC in the context of underlying alcoholic cirrhosis. Age-adjusted mortality rates (AAMRs) provided by CDC WONDER were used directly; no recalculation was performed. Because the data are publicly accessible and deidentified, this study was exempt from Institutional Review Board (IRB) review.

Study population and variables

We included all decedents aged ≥15 years who met the above ICD-10 criteria. Individuals younger than 15 years were excluded due to extremely low case counts and the differing etiologies of pediatric liver disease. Age was grouped into 15-64 years and ≥65 years to allow stable AAMR estimation across the study period; this broader categorization was necessary because finer age stratification would result in suppression of small cells in CDC WONDER, limiting trend assessment.

Race categories were restricted to Black and White individuals because other racial groups had frequent data suppression (counts <10) that prevented reliable reporting. Similarly, females were excluded from stratified analyses because the number of deaths involving both HCC and alcoholic cirrhosis was insufficient to allow stable, non-suppressed age-adjusted rates. These exclusions reflect database limitations rather than conceptual decisions and may result in underrepresentation of known disparities.

Geographic variables included U.S. Census regions (Northeast, Midwest, South, and West), urban vs. rural residence, and state-level mortality data. Socioeconomic region categories were used as provided by CDC WONDER, based on county-level classifications.

Outcomes

The primary outcome was the AAMR for deaths attributed to HCC, with alcoholic cirrhosis recorded as a contributing cause. Secondary outcomes included AAMR stratified by age group, race, census region, urban-rural classification, and state.

Statistical analysis

Temporal trends in AAMR from 1999 to 2020 were assessed using the Mann-Kendall test, a non-parametric method for detecting monotonic trends without assuming normality or linearity. The Kendall tau coefficient quantified the direction and strength of the association, and statistical significance was defined as p < 0.05.

We selected the Mann-Kendall test because the small annual counts for several subgroups limited the feasibility of regression models requiring distributional assumptions. However, we acknowledge that this test does not estimate effect magnitude or account for nonlinear trends; future studies using regression-based models with covariate adjustment may provide more robust trend characterization. Analyses were performed using IBM SPSS Statistics for Windows, Version 27 (released 2019; IBM Corp., Armonk, NY, USA).

## Results

**Figure 1:**
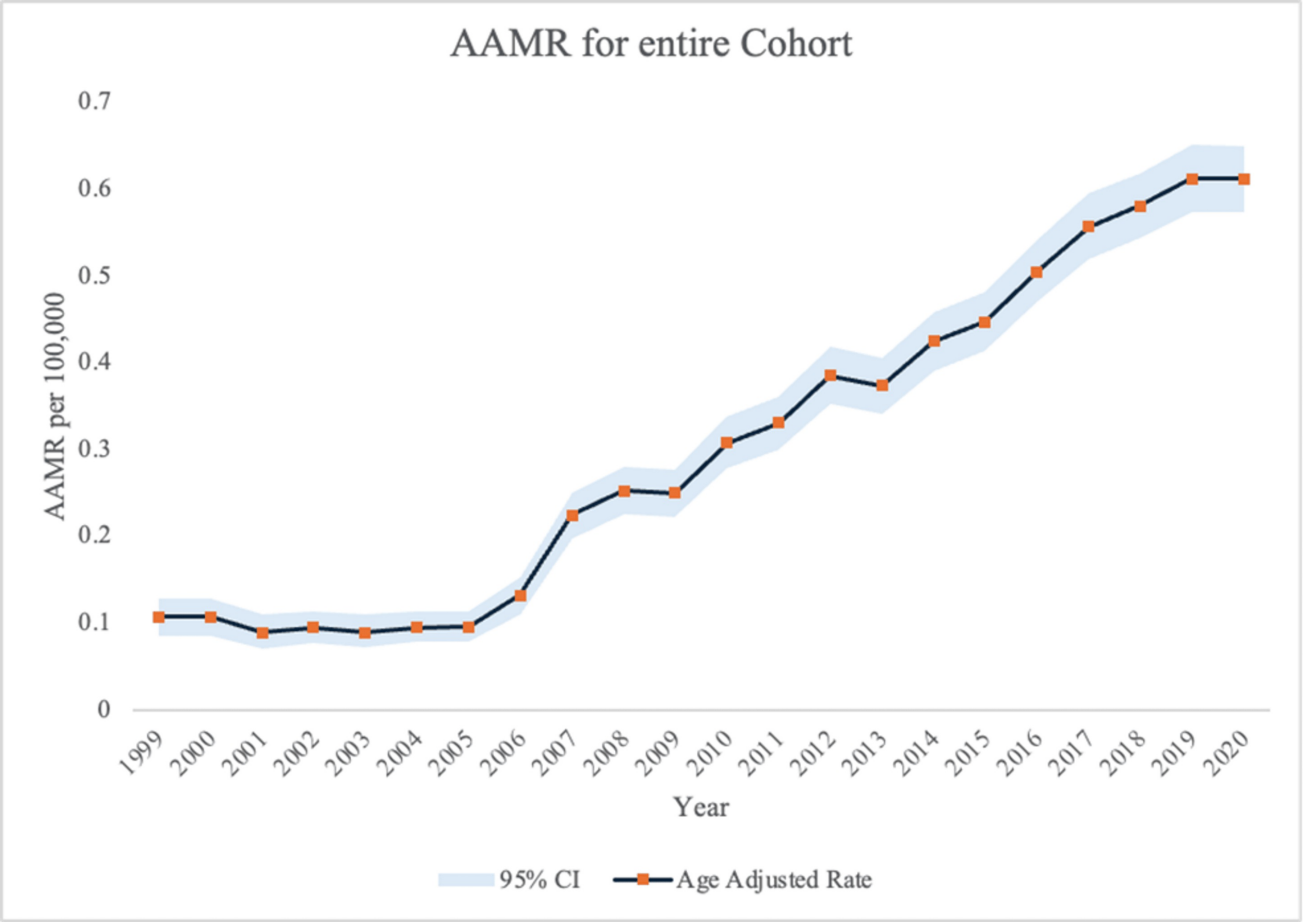
AAMR from HCC in patients with alcoholic liver disease from 1999 to 2000 AAMR, age-adjusted mortality rate; HCC, hepatocellular carcinoma

Between 1999 and 2020, 9,837 adult males diagnosed with alcoholic cirrhosis died from HCC. The AAMR significantly increased (𝜏 = 0.878, p < 0.001) from 0.107 per 100,000 people (95% CI: 0.086-0.128) in 1999 to 0.612 per 100,000 people (95% CI: 0.574-0.65) in 2020. The overall AAMR for this period was 0.338 per 100,000 (95% CI: 0.332-0.345) (Table 1 and Figure 1). The national rate nearly sextupled over the decades, showing a major upward trend. 

**Table 1:** Mean age-adjusted rates of mortality due to HCC in patients with alcoholic liver cirrhosis HCC, hepatocellular carcinoma

Cohort	Male (95% CI)
All	0.338 (0.332-0.345)
White individuals	0.338 (0.331-0.346)
Black individuals	0.348 (0.326-0.369)
Northeast	0.21 (0.198-0.222)
Midwest	0.275 (0.261-0.288)
South	0.319 (0.308-0.329)
West	0.522 (0.504-0.540)
Age 15-64 years	0.241 (0.235-0.247)
Age ≥65 years	0.848 (0.82-0.876)

Among all deaths, 36.7% occurred in inpatient medical facilities, followed closely by 36.3% that occurred at home. An additional 12.9% of individuals died in hospice facilities, 7.6% in nursing homes, and 4.2% in settings classified as “other.” Only 2.1% of deaths occurred in outpatient or emergency department facilities, and the place of death was unknown for 0.1% of cases.

Age stratification

**Figure 2:**
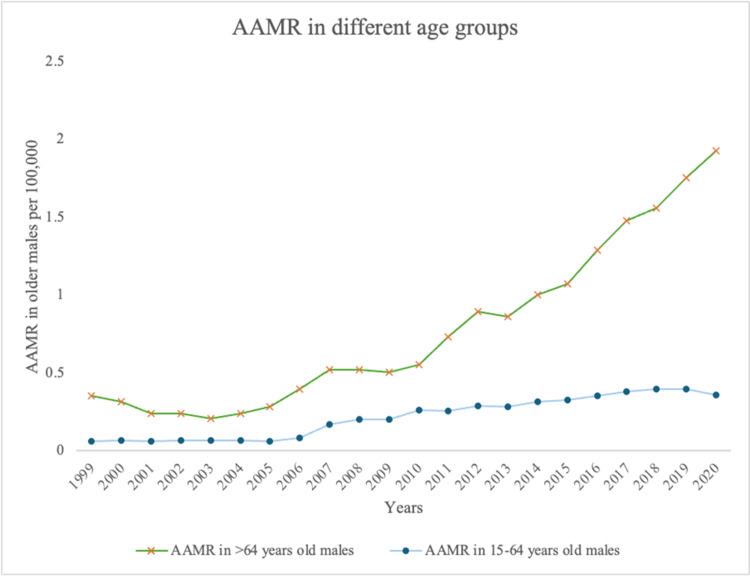
AAMR stratified according to different age groups (15-64 vs. ≥65 years) from 1999 to 2020 AAMR, age-adjusted mortality rate

Among young men (ages 15-64), the AAMR rose markedly from 0.06 (95% CI: 0.046-0.077) in 1999 to 0.36 (95% CI: 0.328-0.392) in 2020, representing a statistically significant upward trend (𝜏 = 0.887, p < 0.001). Over the entire study period, the overall AAMR for this group was 0.241 (95% CI: 0.235-0.247) (Table 1 and Figure 2).

Older men (≥65 years) showed a similarly significant increase in AAMR (𝜏 = 0.852, p < 0.001), rising from 0.352 (95% CI: 0.258-0.470) in 1999 to 1.927 (95% CI: 1.757-2.098) in 2020. Across the full study period, their overall AAMR was 0.848 (95% CI: 0.820-0.876) (Table 1). Throughout all years, older men consistently had significantly higher AAMRs than younger men (p < 0.001).

Racial stratification

**Figure 3:**
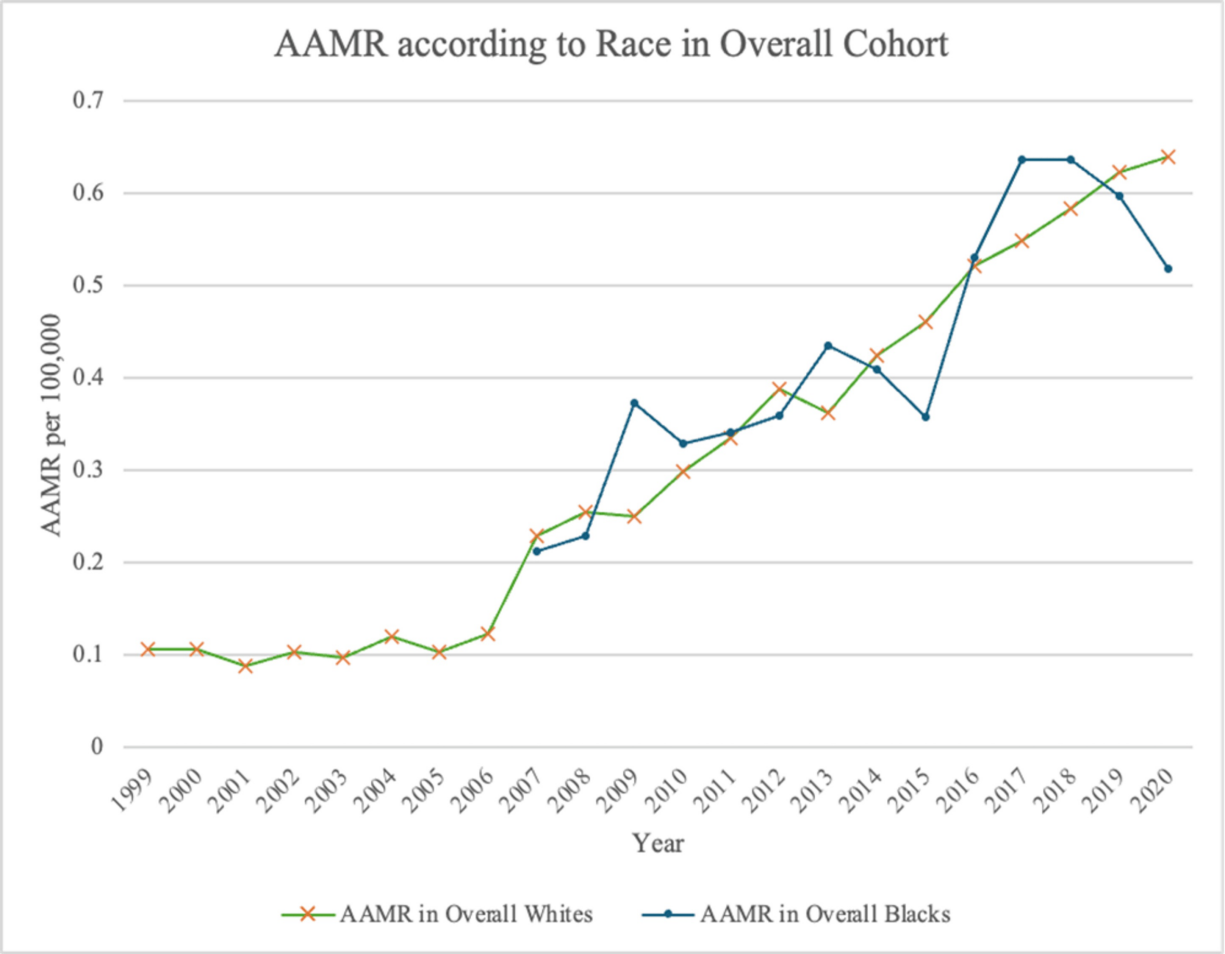
AAMR stratified according to race (data for Black men was unavailable before 2007) from 1999 to 2020 AAMR, age-adjusted mortality rate

Among White men, the AAMR increased significantly over time (𝜏 = 0.891, p < 0.001), rising from 0.107 in 1999 to 0.640 in 2020, with an overall AAMR of 0.338 (95% CI: 0.331-0.346). Black men demonstrated a similar upward trend, with the AAMR increasing from 0.213 in 2007 to 0.518 in 2020, also reaching statistical significance (𝜏 = 0.692, p = 0.001). Their overall AAMR was 0.348 (95% CI: 0.326-0.369) (Table 1). Despite these increases, there was no statistically significant difference in AAMR between White and Black men across the study period (p = 0.055) (Figure 3).

The AAMRs of Black men were either deemed unreliable for certain years (1999-2006) by the CDC or suppressed for privacy protection.

Geographic stratification

**Figure 4:**
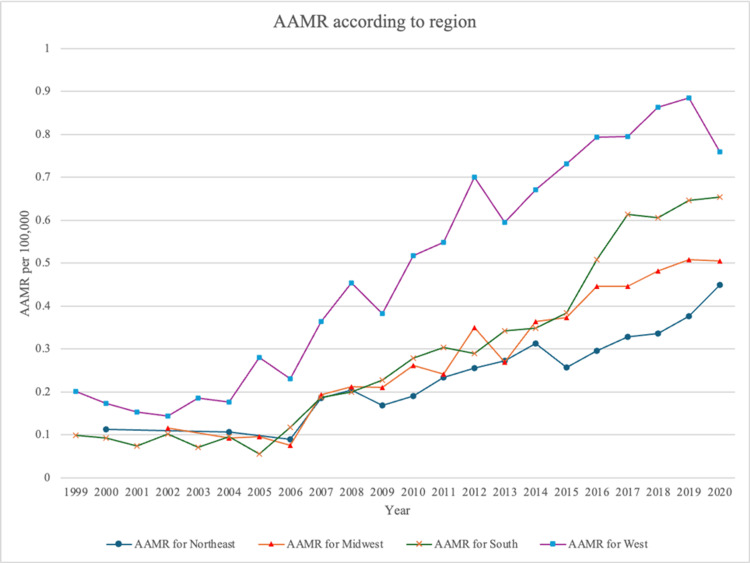
AAMR stratified according to geographical area from 1999 to 2020 AAMR, age-adjusted mortality rate

**Figure 5:**
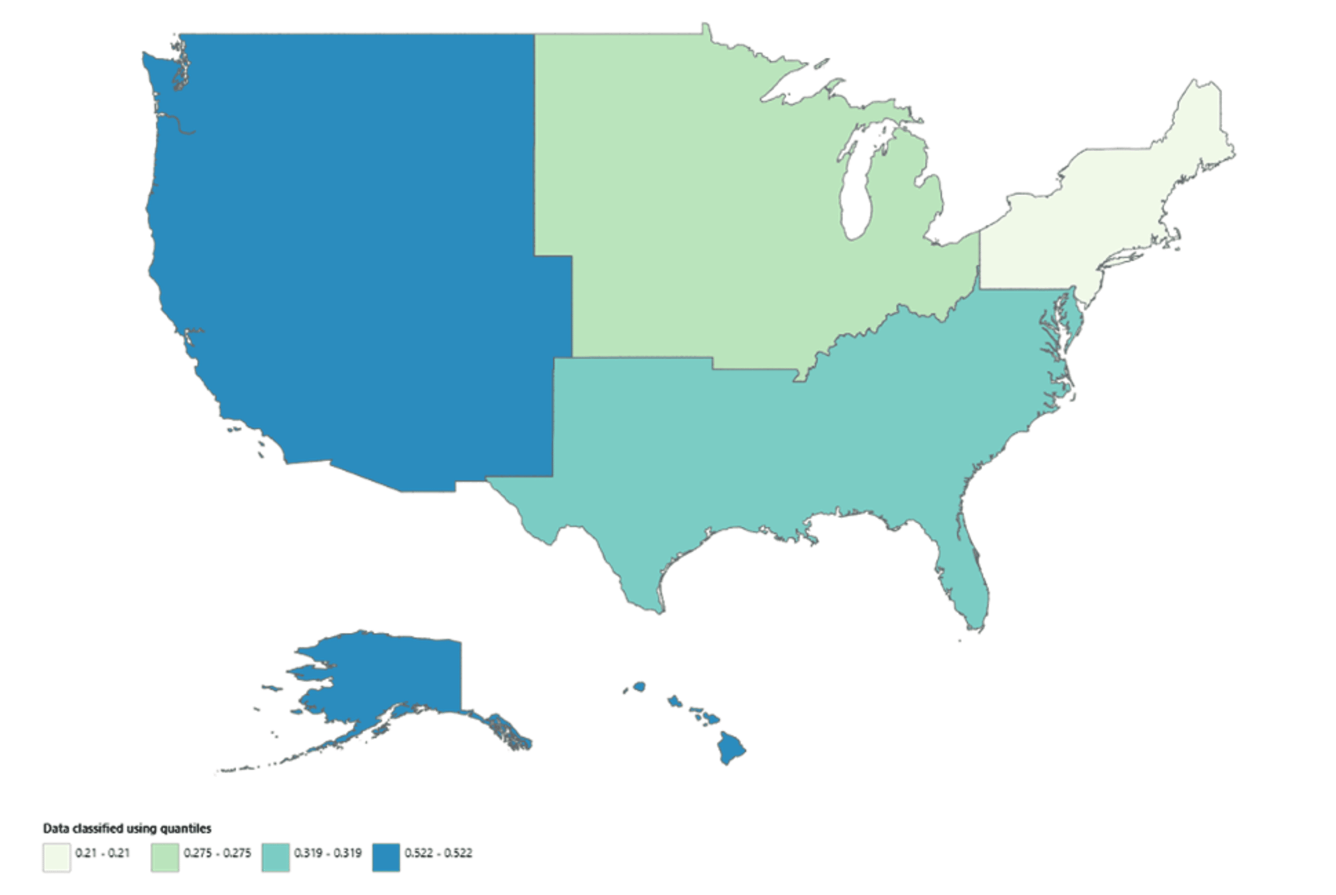
Census region map of trends in AAMR from HCC in patients with alcoholic liver disease from 1999 to 2020 AAMR, age-adjusted mortality rate; HCC, hepatocellular carcinoma

Across U.S. Census regions, the West had the highest overall AAMR at 0.522 per 100,000 people (95% CI: 0.504-0.540), followed by the South at 0.319 (95% CI: 0.308-0.329). The Midwest and Northeast had similar AAMRs (Table 1). All four regions demonstrated significant upward trends in mortality from 1999 to 2020 (𝜏 = 0.879 for the Midwest; 𝜏 = 0.853 for both the South and West; 𝜏 = 0.868 for the Northeast; p < 0.001 for all) (Figures 4-5).

**Figure 6:**
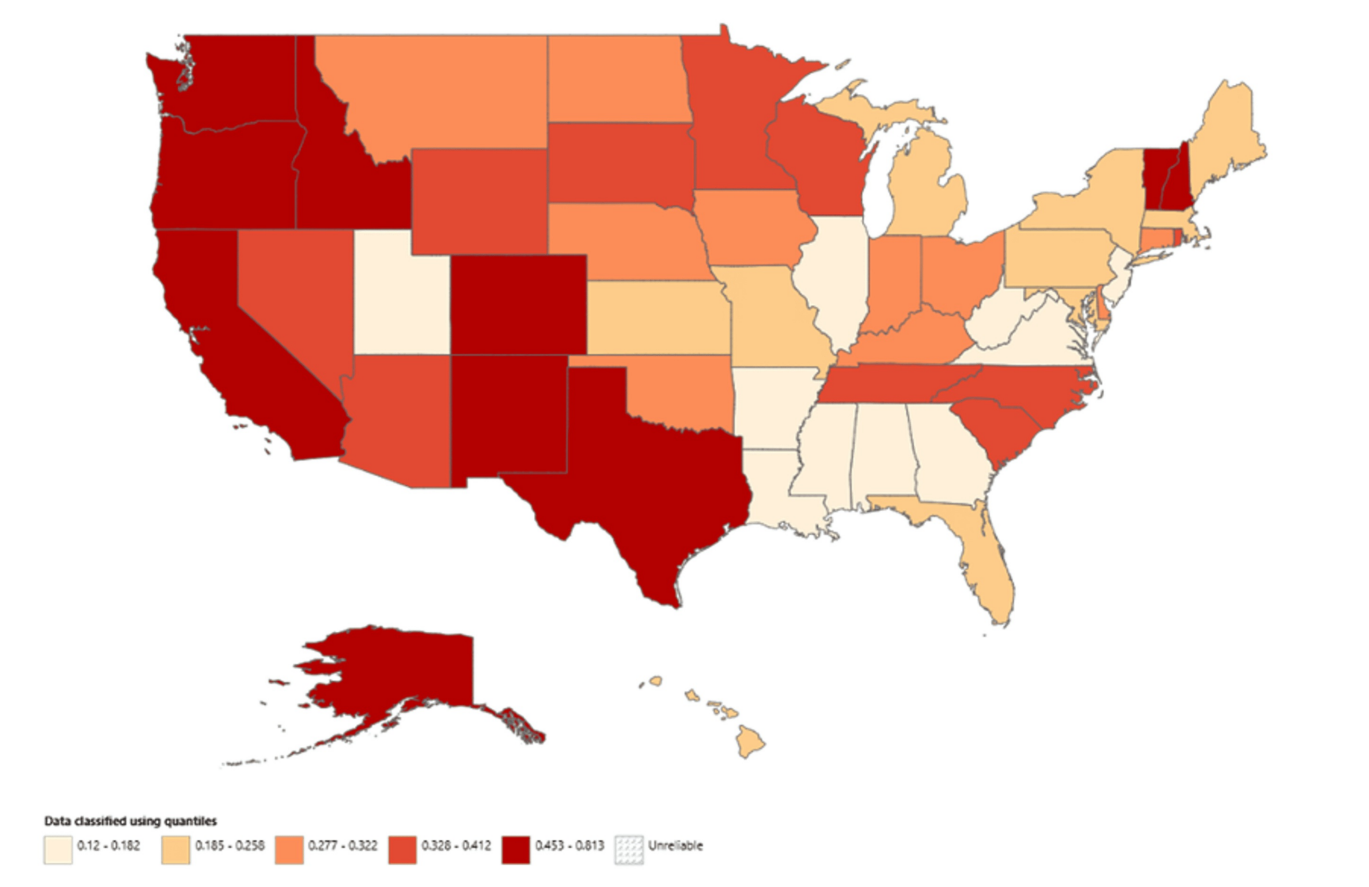
State map of trends in AAMR from HCC in patients with alcoholic liver disease from 1999 to 2020 AAMR, age-adjusted mortality rate; HCC, hepatocellular carcinoma

State-level analysis revealed that New Mexico (AAMR: 0.813; 95% CI: 0.685-0.941), Vermont (AAMR: 0.790; 95% CI: 0.588-1.039), and Oregon (AAMR: 0.737; 95% CI: 0.651-0.823) had the highest mortality rates over the study period. In contrast, the lowest AAMRs were observed in Mississippi (0.132; 95% CI: 0.091-0.185), Utah (0.132; 95% CI: 0.085-0.197), and New Jersey (0.120; 95% CI: 0.097-0.144) (Figure 6). The District of Columbia was not included in this analysis because its overall AAMR was deemed too unreliable by CDC WONDER.

Socioeconomic stratification

In urban areas, the AAMR rose from 0.118 (95% CI: 0.095-0.146) in 1999 to 0.617 (95% CI: 0.575-0.659) in 2020, representing a statistically significant upward trend (𝜏 = 0.894, p < 0.001). Across the entire study period, the overall AAMR for urban regions was 0.347 per 100,000 people (95% CI: 0.339-0.354) (Table 1).

**Figure 7:**
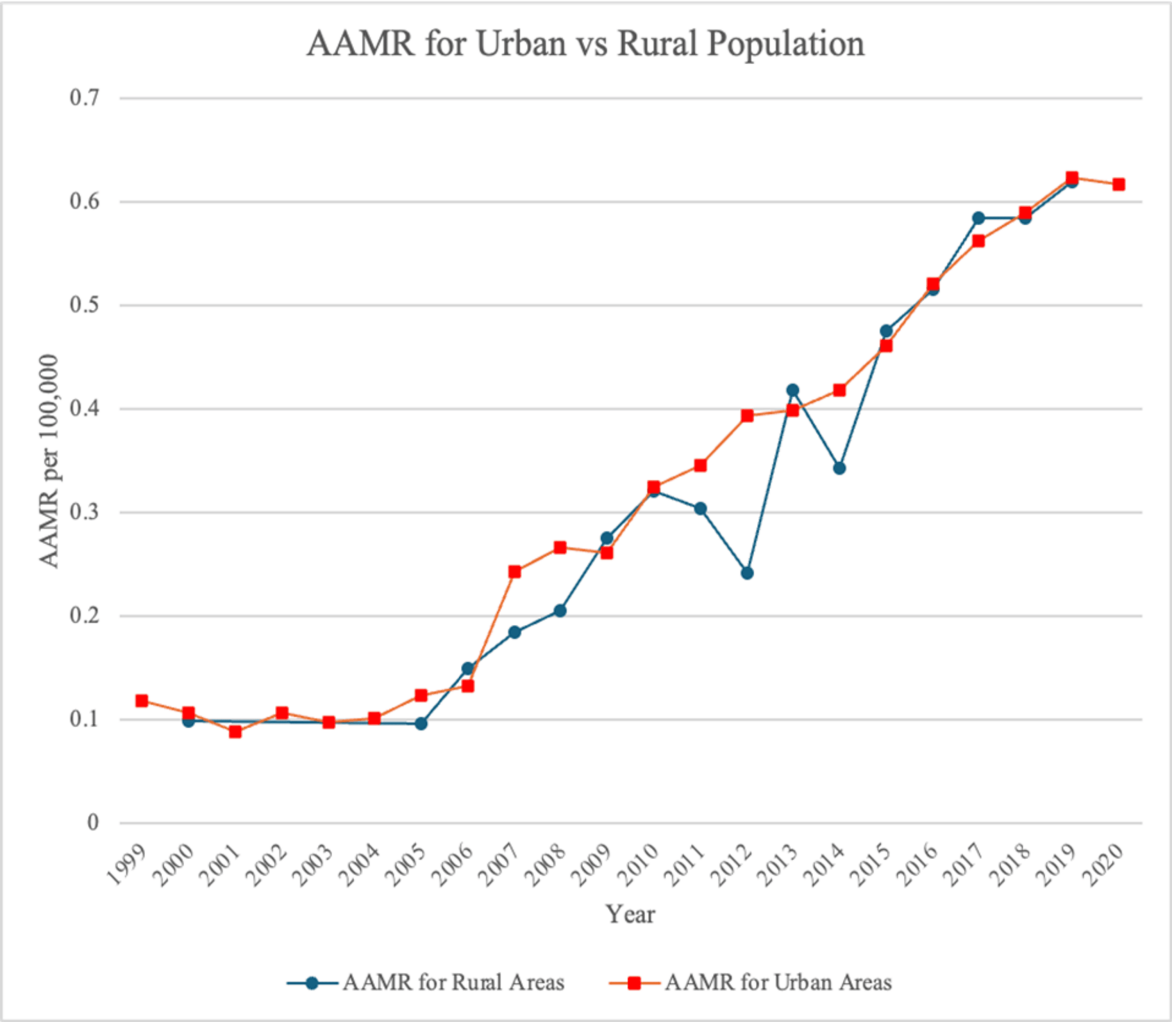
AAMR stratified according to socioeconomic factors from 1999 to 2000 AAMR, age-adjusted mortality rate

Rural men also experienced a significant increase in AAMR (𝜏 = 0.893, p < 0.001), rising from 0.099 (95% CI: 0.060-0.154) in 2000 to 0.620 (95% CI: 0.524-0.716) in 2020. Over the full study period, the overall AAMR for rural men was 0.274 (95% CI: 0.260-0.288) (Table 1). However, when compared with urban men, the AAMRs among rural men did not differ significantly (p = 0.094) (Figure 7). For rural men, since the deaths were too low in certain years, the AAMR was deemed unreliable for those years.

## Discussion

Our analysis revealed a significant rise in alcohol-related HCC mortality among males, aligning with global trends reported by the WHO and the Global Burden of Disease (GBD). Over the study period, 9,837 deaths were attributed to HCC linked to alcohol-associated cirrhosis. Although chronic liver disease (CLD) and its complications impose a substantial and growing global burden, regional disparities persist due to varying sociodemographic conditions. This pattern is particularly relevant for HCC associated with alcoholic cirrhosis, where diagnosis relies heavily on self-reported alcohol use, contributing to potential underestimation and misclassification [8,9].

Within the U.S., our findings mirror these trends, showing a growing disease burden, especially in older men, who consistently exhibited higher AAMRs compared with younger men (p < 0.001). These differences may reflect varying access to healthcare, lifestyle and metabolic risk profiles, and disparities in public health implementation.

This age-related trend also parallels risk projections from the GBD framework, which identifies alcohol use as a major driver of rising DALYs (Disability-Adjusted Life Year) among individuals over 25, particularly those affected by ALD [10]. Our findings support these predictions, as older adults demonstrated the steepest increases in mortality. The disparities observed across age groups likely arise from cumulative lifetime alcohol exposure, interacting with other modifiable risk factors.

Extending beyond age, geographic variation further underscores the complexity of alcohol-related HCC mortality. The Western, Southern, Midwestern, and Northeastern regions of the U.S. all demonstrated consistent increases in mortality. These trends highlight the need for broader preventive strategies, particularly given rising alcohol consumption in developing regions [11]. Variability in healthcare systems contributes to this disparity; countries with robust national prevention programs have achieved better outcomes, whereas the U.S. continues to struggle with the widespread implementation of such strategies [12,13].

One factor that may amplify these regional and demographic trends is the persistently low surveillance rate among patients with alcohol-associated cirrhosis. Limited disease awareness, competing clinical priorities, and provider concerns about adherence all contribute to the underutilization of recommended screening practices [14-16]. This is especially concerning, given that HCC risk varies more than 30-fold depending on patient risk profiles, yet current guidelines continue to rely on a uniform biannual ultrasound strategy for all cirrhotic individuals [17]. As emerging evidence suggests, a shift toward individualized, risk-stratified surveillance may better address this variability [18].

Further emphasizing the need for personalized care, risk factors such as diabetes, smoking, and hepatic decompensation significantly influence HCC development in alcohol-associated cirrhosis, with diabetes showing the strongest association [19]. These findings reinforce the importance of proactive counseling, targeted risk factor modification, and continuous monitoring. Although Hagström et al. question the utility of surveillance once HCC is established, Huang et al. highlight the potential to prevent HCC through early intervention in at-risk patients [19].

Beyond clinical factors, our results also raise concerns about potential underreporting and data limitations. Inaccuracies in death certification, limited disease awareness, and insufficient diagnostic capacity - particularly in low-socio-demographic index (SDI) areas - may contribute to underestimated HCC mortality [20,21]. Addressing these gaps will require enhanced diagnostic infrastructure, improved public health reporting, and better integration of SDOH into existing surveillance systems.

Despite the insights generated, the study has several limitations that must be acknowledged. Its retrospective, observational design is subject to inherent biases. Our reliance on aggregate CDC WONDER data and ICD-10 codes may introduce misclassification error, while the absence of SDOH variables in the database limits our ability to contextualize mortality patterns across populations [22]. These limitations are especially relevant, given the substantial influence of education, socioeconomic status, and healthcare access on disease outcomes.

Finally, the study’s focus on male patients reflects data constraints rather than disease biology. Female mortality data were insufficient to ensure reliable trend estimation, and similar limitations affected analyses of Black men and rural populations due to frequent data suppression. These gaps highlight the need for improved data completeness and more representative population-level reporting, especially when selecting inclusion criteria in future studies. The need for improved screening and prevention tools is also growing and must be focused upon with a fine eye. 

## Conclusions

The rising mortality from HCC associated with alcoholic cirrhosis in the United States remains a significant public health concern. Our analysis demonstrated steadily increasing AAMR across age groups, races, and regions, with older men and Western states carrying the highest burden. To our knowledge, this study provides one of the first comprehensive national evaluations of age-, race-, and state-level disparities in alcohol-related HCC mortality, adding important insight to the existing literature.

Reducing this trend will require earlier identification of at-risk individuals, broader implementation of alcohol screening and brief interventions, and stronger integration of HCC surveillance into routine care. Public education efforts promoting alcohol risk awareness and liver health, combined with region-specific, data-driven strategies that incorporate SDOH, will be essential to improving access to evidence-based care and reducing mortality disparities nationwide.
